# Does CT Reduce the Rate of Negative Laparoscopies for Acute Appendicitis? A Single-Center Retrospective Study

**DOI:** 10.25122/jml-2019-0099

**Published:** 2020

**Authors:** Pedro de J Wagner, Muthana Haroon, Stefan Morarasu, Emmanuel Eguare, Osama Al-Sahaf

**Affiliations:** 1. Department of Surgery, Naas General Hospital, Naas, Ireland; 2. Trinity College, Dublin, Ireland

**Keywords:** Acute appendicitis, laparoscopy, CT scan

## Abstract

In surgical practice, surgeons request CT scans to rule out acute appendicitis, even in young patients. We aimed to assess the feasibility of using a CT scan to reduce the rate of negative laparoscopies in patients younger than 40 with equivocal signs of acute appendicitis.

Therefore, we conducted a retrospective observational study on the patients admitted with a provisional diagnosis of acute appendicitis. Patients younger than 40 and with the Alvarado score between 3 and 6 were included. These were divided into two groups: those who had or did not have a CT scan. Each group was further subdivided into patients that had a laparoscopy and those that did not.

Out of 204 patients included in the study, 16% were included in the CT group, and 84% in the non-CT group. 71.9% of the patients that underwent a CT scan had appendicitis and underwent an appendectomy. Five patients with a normal CT scan had appendectomy due to persistent signs of acute appendicitis. The histopathology of the 23 patients with positive CT was positive, and 3 of the 5 patients with negative CT that underwent appendectomy had positive histology results. The negative appendectomy rate for patients that had preoperative CT is 7.14% compared to 32.4% in patients without preoperative CT.

The rate of negative laparoscopy in patients younger than 40 years old that undergo preoperative CT is significantly lower with a p-value of .00667.

## Introduction

Acute appendicitis is the most common acute surgical emergency worldwide, with a rate of about 10 per 10000 a year in the US. The peak age of incidence ranges from 10-20 years, with the peak range of incidence of 16 to 40 years in the adult population [1-3]. The lifetime risk is about 7-8%, and it is most common in males, but females of childbearing age are twice more likely to undergo laparoscopy and appendicectomy due to a broader range of differential diagnosis [4].

The diagnosis of acute appendicitis can often be made on clinical bases, and clinical scoring systems such as the Alvarado score can aid diagnosis. While CT scan is not always required to confirm the diagnosis, current evidence recommends routine CT scan for patients 50 and older with right iliac fossa (RIF) pain in the diagnostic workup, to rule out a possible neoplasm [12]. Often, however, clinical diagnosis is challenging, particularly in early presentation, and further investigation such as CT should be considered even in younger patients [13, 14]. This leaves the surgeon trapped in a dilemma whether to proceed with laparoscopy and ignore the risks of a potentially unnecessary procedure, or proceed with a CT scan, exposing the young patient to the risks of radiation [15], only to find that laparoscopy is needed anywise, either due to positive or inconclusive CT findings [16].

In the USA, there is indiscriminate use of CT scans in nonpregnant patients with a suspicion of acute appendicitis, with estimates of more than 95% of patients undergoing scan before surgery, in contrast to about 13% in Europe according to studies [17]. According to some literature, this indiscriminate use of CT scans has significantly reduced the rate of negative appendicectomy, while others refute this [17].

The body of evidence available on this research topic supports both arguments, with higher evidence supporting that routine CT for suspected acute appendicitis decreases the rate of negative laparoscopy. However, most literature focuses entirely on CT, laparoscopy, and histology outcomes without taking into consideration the appropriateness of the clinical indication for CT, laparoscopy, or appendicectomy in the first place. Furthermore, most of the studies do not focus on specific demographics, thus increasing limitations since the incidence of appendicitis has a peak age range.

This research focuses on adult patients 40 years or younger, which is the age range with the highest incidence of suspected and confirmed acute appendicitis. The study also focuses on patients with equivocal signs of acute appendicitis, defined here as Alvarado scores 3-6.

## Material and Methods

The study design is a retrospective, non-randomized case-control trial. It was conducted at the Department of Surgery of Naas General Hospital, Ireland. After approval by the local Ethics committee, data from all the patients admitted in this hospital with suspected acute appendicitis from January 2015 to December 2017 was collected using Hospital In-Patient Enquiry (HIPE).

Inclusion criteria:

• Patients with suspected acute appendicitis aged 40 years or younger
• Alvarado score between 3 and 6

Patients with Alvarado scores 3-6 were selected and divided into two groups: patients that had a CT scan done and patients that did not get a CT scan. The group with a CT done had their management guided thoroughly, or in part, by the scan results. For the ones that had no CT done, management was based mainly on clinical findings. The histology results, CT scan reports, and relevant laparoscopy notes (if laparoscopy was performed) were collected for each patient, and any history of re-admission with the same complaints was also recorded. This final data were entered into an Excel file under gender, age, CT results, laparoscopy, and histology outcomes. A standardized form was used during data collection. The patients’ medical record numbers (MRN) were coded during data analysis in order to maintain the patient's anonymity and confidentiality, Statistical analysis was done using IBM SPSS. Categorical data such as histopathology results were compared using the Chi-squared test, whereas variables were compared using the t-test. Data was presented in numbers and percentages, and statistical significance was indicated by a p-value of <0.05. A true positive result for patients in the CT scan group means that they had positive findings on CT and histopathology. A true positive result of patients in the non-CT group means positive clinical findings confirmed by positive histopathological results. In our department, all patients that underwent laparoscopy also underwent appendectomy, regardless of intraoperative findings.

## Results

**Figure 1:**
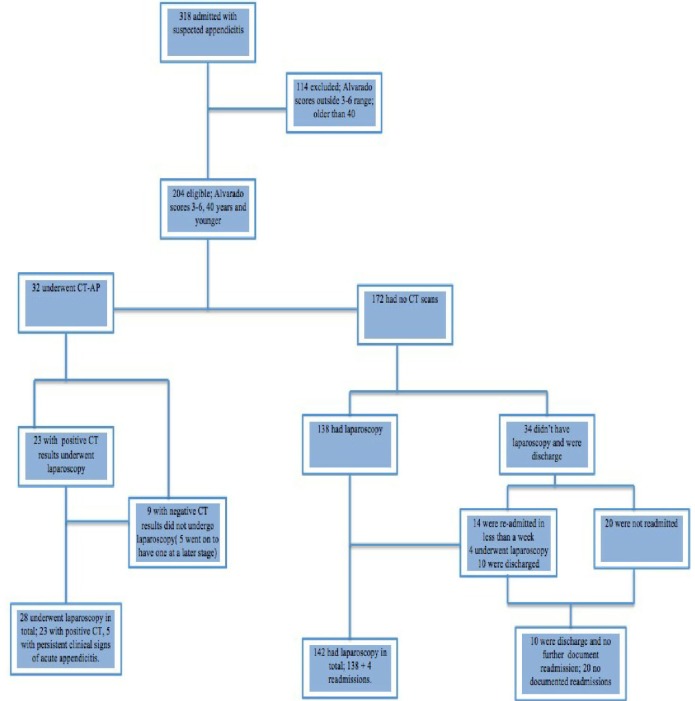
Cohort Overview.

A total of 318 patients were admitted with suspected acute appendicitis between January 2015 to December 2017. Out of these, 80 patients were excluded for being older than 40 at the time of admission. A further 34 patients were excluded due to either having Alvarado scores outside of the range or scores that could not be ascertained (Figure 1).

Out of the 204 patients, 172 (84%) underwent preoperative CT scans, and 32 (16%) did not. Almost twice as many females underwent CT scans than males, with 65.6% of the scanned patients being females and 34.4% being male. This difference is statistically significant, with a p-value of 0.026. The mean age of the patients who got a CT scan was 33.06 with SD +/- 6.584, while the mean age for people who did not get a CT scan was 27.08 with SD +/- 6.801. The t-value is .000, which means there is a statistically significant difference in the age between the two groups. Overall, more males presented with suspected acute appendicitis than females, 107 (52%) and 97 (48%), respectively (Table 1).

**Table 1:** Patients’ demographics.

Groups	No. of patients	Female	Male	Mean age
**No CT**	172	76 (44.2%)	96 (55.8%)	23
**CT**	32	21 (65.6%)	11 (34.4%)	33

The CT findings were predominantly simple appendicitis, with 20 patients out of 32 having this finding (62.5%). Complicated appendicitis such as perforated appendix and appendiceal mass occurred in 3 patients (9.5%). The CT was normal in 8 (25%) patients, and 1 (3.1%) patient had a finding classified as other pathology, which did not require surgical input.

Appendectomy was performed on 142 patients out of the 172 of the non-CT group (82.6%). Initially, 34 patients out of the 142 were managed conservatively and observed for an average period of 72 hours then discharged. Out of the 142 patients that had an appendectomy, 45(31.7%) had a normal appendix on histology, 91 (64.1%) had acute non-complicated appendicitis, and 5 (3.5%) had acute complicated appendicitis. Crohn's disease was found in 1 (0.7%) patient (Table 2).

**Table 2:** Histopathology findings.

Groups	Histology Results			
Simple appendicitis	Complicated appendicitis	Crohn’s	Normal appendix
**No CT**	91 (64.1%)	5 (3.5%)	1 (0.6%)	45 (31.7%)
**CT**	20 (71.4%)	6 (21.4%)	0	2 (7.14%)

As for the patients in the CT group, appendectomy was performed in 28 cases (87.5%); 23 based on positive CT findings and 5 based on the persistent clinical picture of appendicitis despite negative CT findings. Out of these, 20 out of 28 (71.4%) had non-complicated appendicitis, 6 out of 28 (21.4%) had complicated appendicitis, and 2 (7.1) had a normal appendix on histology (Table 2). The four patients that had no appendicectomy from the CT group were discharged after conservative management or had a further evaluation for another diagnosis. There were no recorded re-admissions in this group (Figure 1).

Regarding the sensitivity and specificity of CT versus clinical evaluation alone, the clinical evaluation showed a 95.8% sensitivity based on the true positive rate of 92 patients and a false-negative rate of 4. The specificity was 39.7% based on the true negative rate of 30 patients and a false positive rate of 46. CT, on the other hand, showed an 86.4% sensitivity based on the true positive rate of 19 and a false-negative rate of 3. The specificity was found to be 100% based on a true negative rate of 6 and no false-positive findings (Table 3). False-negative in the CT group was defined as patients who had negative findings on CT but underwent laparoscopy based on clinical suspicion, and subsequent histology confirmed the acute appendicitis diagnosis. False-negative in the non-CT group was defined as patients who had clinical exam inconsistent for acute appendicitis (Alvarado less than 3) but underwent diagnostic laparoscopy due to non-resolving symptoms and subsequent histology confirmed acute appendicitis. The false-negative rate was 3 (9.4%) in the CT group and 4 (2.3%) in the non-CT group.

**Table 3:** Sensitivity and specificity of clinical evaluation versus CT scan in equivocal signs of appendicitis.

Groups	False negative	False positive	True negative	True positive	Sensitivity	Specificity
**No CT**	4 (2.3%)	46 (26.7%)	30 (0.6%)	92 (53.5%)	95.8%	39.7%
**CT**	3 (9.4%)	0	5 (18.8%)	23 (71.9%)	88.4%	100%

For the 142 patients without CT that underwent appendectomy, 46 had negative histopathology findings, giving a rate of 32.4% negative laparoscopic appendectomy. Out of the patients that had CT and appendectomy, 2 had negative histopathological findings, giving a rate of 7.14%. The positive laparoscopy rate was 67.6% for the patients in the non-CT group and 92.6% for the patients in the CT group. Using this data, the Chi-square test was used to calculate if there is a significant difference between the two groups, CT and non-CT. This showed a p-value of 0.00667, which is significant according to the reference value of p<0.05 (Table 4). When comparing the odds ratio, patients who undergo laparoscopic appendectomy based on CT confirmation are six times more likely to have histology-confirmed appendicitis than those who undergo surgery based only on clinical examination without CT (OR = 0.1641, 95% CI 0.0373-0.7218, Z statistic 2.391, p = 0.0168) (Tables 3, 5).

**Table 4:** Rate of negative and positive laparoscopy.

Groups	Laparoscopy findings	
Negative laparoscopy	Positive laparoscopy
No CT	46 (32.4%)	96 (67.6%)
CT	2 (7.14%)	26 (92.85%)

**Table 5:** Contingency table on comparing histologically-confirmed appendicitis chances in patients who have had or not a preoperative CT.

	Positive Histology (acute appendicitis)	Negative Histology	Total
CT confirmed Laparoscopic Appendectomy	26	2	28
No CT, clinically diagnosed Laparoscopic Appendectomy	97	45	142

## Discussion

This research shows that a CT scan decreases the rate of negative appendectomy, rejecting the null hypothesis. The negative appendectomy rate for patients that did not have a CT scan was 32.4% compared to 7.14% for patients that had a CT scan. This represents a relative risk of approximately 4.5 times of getting negative laparoscopy if CT is not done in such a group of patients. However, it is crucial to mention the gender discrepancy seen in this research. Although slightly more male presented with suspected appendicitis, women were more likely to undergo a CT and laparoscopy than men. This discrepancy is mostly due to the broader range of differential diagnosis for acute appendicitis in women compared to men, particularly gynecological pathology [2]. As part of our internal protocol, 98% of women had an ultrasound (US) and other investigations to exclude gynecological issues. The US is excellent in ruling out gynecological conditions, but its sensitivity and specificity for acute appendicitis are relatively low, 86%, and 81%, respectively, and it is operator-dependent [18].

Another important finding is that a higher percentage of patients that had a CT scan had complicated appendicitis on histopathology, compared to patients that had laparoscopic appendectomy based on a clinical decision only, with 21.4% complicated appendix for the CT group compared to 3.5% for the non-CT group. Half of the complicated appendicitis cases in the CT group was reported as such on the CT, but the other half was reported as uncomplicated appendicitis. Despite a high true positive rate for CT and high overall sensitivity and specificity, it should be noted that the false-negative rate was 9.4%, thus even in patients who have negative CTs, the clinical suspicion should not be neglected.

D’Souza et al. analyzed the cost-effectiveness of routine imaging in suspected acute appendicitis [19]. They looked into histology results, length of hospital stays, and operation time to calculate the costs. The conclusion was that at their institution, there was a negative appendectomy rate of about 22%, which implies costs of roughly 303.699 euros. The routine use of CT would have reduced the costs to 185.690 euros.

Even though the findings here are not unique to this study, as far as we are concerned, there is no current available literature focusing on these specific points, specific age groups, or specific clinical parameters. Since one size does not fit all, to develop adequate guidelines, it is necessary to approach the diagnosis of equivocal acute appendicitis by first determining its likelihood in the first place, which can be done using the Alvarado score. Secondly, the approach should be different in males and females, which are essential conclusions drawn from this research [19,20].

An important reason why surgeons restrain from using CT scans in young patients, especially females of childbearing age, is the exposure to radiation. One approach to decreasing radiation exposure could be to decrease the radiation dose per scan. A randomized controlled trial was conducted by Kyuseok et al. to compare the rate of negative appendicectomy in patients that had preoperative low-dose abdominal CT versus those that had a standard CT. The study results showed negative appendicectomy rates of 3.5% and 3.2% for standard and low-dose CT, respectively [21]. This difference is not significant, and the study concludes that a low radiation dose CT scan was not inferior to the standard CT scan in diagnosing acute appendicitis. There was also no difference in the number of patients that needed additional investigation [21].

According to multiple studies, CT scans decrease not only the rate of negative appendectomy but also the rate of complications [21, 22]. The pattern of increased complication rate observed here might be attributed to delays caused by backlogs in the institutions’ radiology department, delays in the decision to perform a CT caused by observation approach, or a combination of these. A decision to perform CT should be made hastily, and if the decision is to observe the patient, a change or persistence of the clinical picture should prompt laparoscopy.

Improvement of clinical evaluation can better guide decision-making in suspected acute appendicitis, and possibly decrease the rate of unnecessary CT scans or laparoscopy. A systematic review by Ohle at al. [24]) suggests the strict use of the Alvarado score to rule out appendicitis below the value of 5. Above this point up to 6, it would be appropriate to either perform a CT or diagnostic laparoscopy to confirm, whichever can be done sooner.

A significant limitation of this study is the discrepancy (one to five) between the two groups. This might have decreased the power of the study and increased the likelihood of type 2 error. In addition, this data was collected in a single-center, and as much as the results here resonate with those of multiple similar studies, it may not be extrapolated to the general population.

## Conclusion

This study concludes that a CT scan reduces the rate of negative appendectomy and provides a good overview of the challenges faced in diagnosing equivocal acute appendicitis.

## Conflict of Interest

The authors confirm that there are no conflicts of interest.
